# Estrogen synthesized in the central nervous system enhances MC4R expression and reduces food intake

**DOI:** 10.1111/febs.17426

**Published:** 2025-02-18

**Authors:** Takanori Hayashi, Kanako Kumamoto, Tatsuya Kobayashi, Xinfeng Hou, Shizuko Nagao, Nobuhiro Harada, Shinichiro Honda, Yohei Shimono, Eiji Nishio

**Affiliations:** ^1^ Department of Biochemistry Fujita Health University School of Medicine Toyoake Japan; ^2^ Department of Anatomy and Medical Biology Fujita Health University School of Medicine Toyoake Japan; ^3^ Center for Disease Model and Educational Support Fujita Health University Toyoake Japan; ^4^ Department of Regulatory Science, Research Promotion Unit Fujita Health University School of Medical Science Toyoake Japan; ^5^ Reproduction Center, Fujita Health University Haneda Clinic Otaku Japan; ^6^ Department of Molecular Infectiology, Reproductive Medicine Chiba University of Graduate School of Medicine Chiba Japan; ^7^ Department of Biochemistry Fukuoka School of Pharmaceutical Sciences, Fukuoka University Japan; ^8^ Department of Obstetrics and Gynecology Fujita Health University School of Medicine Toyoake Japan

**Keywords:** appetite regulation, energy homeostasis, melanocortin 4 receptor, neuroestrogen

## Abstract

Estrogen is synthesized throughout various tissues in the body, and its production is regulated by the rate‐limiting enzyme aromatase (encoded by the *Cyp19a1* gene). Notably, aromatase is also expressed in central nervous system cells, allowing for localized estrogen synthesis in regions such as the hypothalamus. Estrogens produced within these neurons are referred to as neuroestrogens. In this study, we investigated the role of neuroestrogens in the regulation of appetite through modulation of hypothalamic pathways in OVX, ArKO, and aromatase‐restored mice. Estrogen suppresses appetite by influencing the expression of appetite‐regulating peptides, including POMC and NPY, via MC4R. We explored the direct effects of neuroestrogens, independent from ovarian estrogen, on appetite suppression and the underlying molecular mechanisms. We monitored body weight and food intake and evaluated the expression of *Cyp19a1*, *Mc4r*, and other appetite‐related genes. Our findings indicate that OVX and ArKO mice exhibited increased body weight and food consumption, which correlated with altered expression of *Mc4r* and *Cyp19a1*. Conversely, restoration of *Cyp19a1* expression in a neuron specific manner significantly decreased food intake and increased *Mc4r* expression in the hypothalamus. Furthermore, neuroestrogens enhanced leptin responsiveness. Our results imply that neuroestrogens likely contribute to appetite regulation and may be relevant for body weight reduction.

## Graphical abstract

Estrogen is regulated by the rate‐limiting enzyme aromatase. This study examines how neuroestrogens, estrogen produced in the brain, regulate appetite through the hypothalamus. Using ovariectomized, aromatase knockout, and brain‐specific aromatase‐restored mice, the study found that neuroestrogens suppress appetite by influencing genes like Mc4r. Mice without aromatase showed increased food intake and weight gain, while restoring Cyp19a1 reduced appetite. The findings provide evidence that neuroestrogens play a role in appetite control.

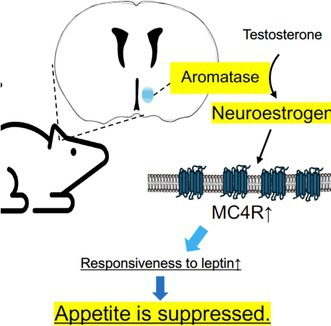

**Abbreviations**
ArKO: aromatase knockout mice
D: diapause
E_2_
: β‐estradiol
ER: estrogen receptor
Esr1: estrogen receptor α
LEPR, Lepr: leptin receptor
Let: letrozole
M: late estrus
Mc4r: melanocortin 4 receptors
NPY, Npy: neuropeptide Y
OVX: ovariectomized mice
OXT, Oxt: oxytocin
OXTR, Oxtr: oxytocin receptor
P&E: early estrus and estrus
POMC, Pomc: pro‐opiomelanocortin
SHAM: sham operation mice
T: testosterone

## Introduction

Estrogen plays a crucial role in the regulation of energy metabolism, appetite suppression, and body weight reduction. Appetite control is intricately balanced by the interplay between appetite‐suppressing and appetite‐enhancing peptides [1, 2, 3, 4]. The appetite suppressor POMC, synthesized in the hypothalamic arcuate nucleus, undergoes metabolism to produce the α‐melanocyte stimulating hormone, which suppresses the appetite by activating MC4Rs [4, 5, 6, 7, 8]. Conversely, appetite‐stimulating peptides such as NPY and agouti‐related peptide act as MC4R antagonists, enhancing appetite by inhibiting appetite‐suppressive functions [4, 5, 6, 9, 10]. Appetite modulation depends on a delicate equilibrium between appetite suppression and enhancement [11], with leptin, secreted by white adipocytes, strongly favoring appetite suppression [9, 11, 12, 13]. MC4R are localized in the hypothalamus, a pivotal region in the brain responsible for appetite regulation, and their expression plays a critical role in appetite inhibition [4, 14, 15]. MC4R binds with melanocortin to inhibit appetite. Activation of this receptor can lead to reduced appetite and enhanced control of meal size. The promoter region of the *Mc4r* gene harbors an estrogen receptor response sequence, which is upregulated by estrogen [16], thereby augmenting sensitivity to appetite‐regulating peptides, such as leptin, through increased *Mc4r* expression [3, 17].

Aromatase, the rate‐limiting enzyme in estrogen biosynthesis, is independently regulated in gonads and other tissues [18, 19, 20]. In the central nervous system, aromatase is notably abundant in the hippocampus, hypothalamus, and amygdala, influencing memory and reproductive behavior [21, 22, 23, 24]. Intraneuronal aromatase impacts short‐lived physiological phenomena like phosphorylation‐modulated fluctuations in neuroestrogens, altering reproductive behavior in male quails [25, 26]. Neuroestrogens possibly participate in various neurophysiological functions, including appetite suppression. However, discerning the appetite‐suppressive effects of estrogens from those of neuroestrogens, which function as endocrine molecules *in vivo*, remains unexplored.

In this study, we investigated the role of neuroestrogens in the modulation of food intake and leptin responsiveness by employing a multifaceted experimental approach. We utilized OVX to create a model devoid of ovarian estrogen production, ArKO that are incapable of synthesizing estrogen due to a systemic absence of aromatase, and a genetically modified mouse model where aromatase expression was reinstated. These models were used to test the hypothesis that neuroestrogens significantly reduce food intake. Subsequently, we expanded our investigation to elucidate the impact of neuroestrogens on *Mc4r* expression, employing both murine models and cultured cellular systems. Furthermore, we explored the comparative leptin sensitivity between ArKO mice and their counterparts in which neuroestrogen production was restored. Our findings indicate that the reestablishment of neuroestrogen synthesis in ArKO mice leads to an enhanced leptin response, surpassing that observed in the original ArKO phenotype. These observations advance our understanding of the intricate roles neuroestrogens play in regulating metabolic functions and energy homeostasis.

## Results

### OVX may increase brain aromatase expression, potentially leading to appetite suppression

**Fig. 1:**
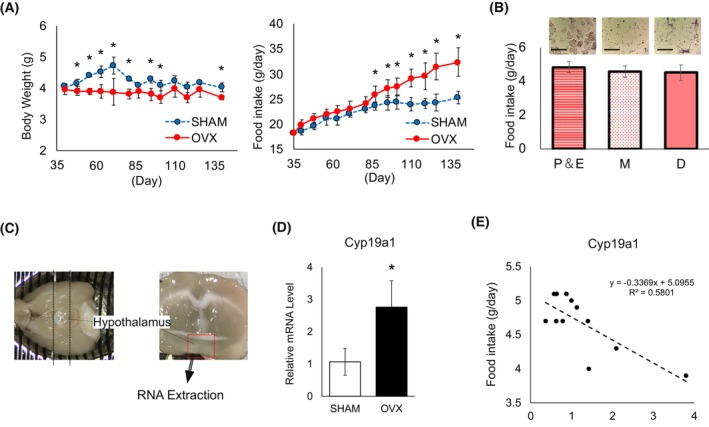
Body weight and food intake in OVX and SHAM mice. (A) Graph showing the body weight and food intake of ovariectomized (OVX, black circles, *n* = 5) and sham‐operated (SHAM, white circles, *n* = 6) female mice from 35 to 140 days of age. (B) Following a 24‐h food intake measurement in 90–95‐day‐old mice, vaginal smears were collected to identify phases of early estrus and estrus (P&E, *n* = 4), late estrus (M, *n* = 4), and diapause (D, *n* = 4). The scale bar (black bar) represents 50 μm. (C) Hypothalamus sampling. First, brains are collected from mice refluxed in ice‐cold PBS, and the brains are sliced at a thickness of 2 mm (black dotted line) to include the hypothalamus. Then, tissue pieces were cut into 1 mm long and 1 mm wide (red dotted line). (D) Cyp19a1 expression in the hypothalamus (*n* = 12). (E) Measurement of mRNA levels of Cyp19a1 in the hypothalamus after a 24‐h food intake assessment in 90–95‐day‐old mice. Statistical analysis was performed using one‐way ANOVA to evaluate differences among the groups. Tukey's *post hoc* test was conducted to identify pairwise differences between the groups (A, B). For pairwise comparisons between two groups, Student's *t*‐test was used (D). To evaluate the relationship between variables, Pearson's correlation coefficient was calculated (E). Results are presented as mean ± standard deviation (SD), and statistical significance was defined as *P < 0.05. All analyses were performed using spss.

OVX mice, in which the ovaries were removed before menarche (age: 28–30 days), were generated to eliminate the effects of ovarian estrogen secretion. SHAM mice, without ovariectomies, served as controls. Changes in body weight and food intake were monitored from after ovarian surgery (35 days of age) until 140 days of age. The OVX group exhibited greater body weight gain compared to the SHAM group from 95 days of age onward (95‐day‐old SHAM vs. OVX: 23.7 ± 0.7 g vs. 26.0 ± 1.6 g; *P* < 0.001). Conversely, food intake tended to decrease immediately after ovariectomy, with a significant decrease observed after 49 days of age (95‐day‐old SHAM vs. OVX: 4.3 ± 0.15 g vs. 3.8 ± 0.13 g; *P* < 0.05) (Fig. 1A).

To explore whether differences in appetite influenced by the menstrual cycle in mice contributed to variations between the OVX and SHAM groups, we assessed menstrual cycles and food intake in 20 mice. Following food intake measurement, PBS was injected into the vagina under anesthesia, and vaginal smears were examined under a microscope. The mice were classified into three groups: proestrus and estrus phase (P&E), metestrus phase (M), and diestrus phase (D). Our analysis revealed insignificant differences among the three groups (Fig. 1B).

To investigate the effect of OVX on aromatase expression in the nervous system, we dissected the hypothalamus of female mice aged 90–95 days (*n* = 5; SHAM/OVX) after recording their food intake. The hypothalamus was sectioned into 2 mm^3^ (2 × 1 × 1 mm) cubes to measure mRNA levels of *Cyp19a1* (Fig. 1C). A significant increase in *Cyp19a1* levels was observed in the OVX mice (Fig. 1D). Furthermore, food intake in mice correlated with *Cyp19* expression in the hypothalamus (Fig. 1E).

### Correlation between Cyp19a1 and appetite regulators in the mouse hypothalamus

**Fig. 2:**
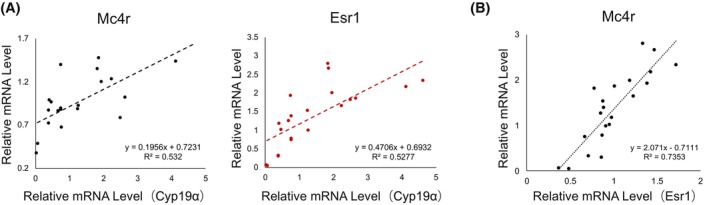
Correlation of *Cyp19* with *Mc4r* and *Esr1* mRNA levels in female mice. (A) Analysis of the correlation between mRNA levels of *Cyp19* with *Mc4r*, and *Cyp19* with *Esr1* in the hypothalamus of mice aged 13–15 weeks. (B) Examination of the relationship between *Esr1* and *Mc4r* mRNA quantities. To evaluate the relationship between variables, Pearson's correlation coefficient was calculated. Statistical significance was defined as *P* < 0.05. All analyses were performed using spss.

To explore the involvement of neuroestrogens in appetite suppression, we examined the expression levels of *Cyp19a1* and the mRNA of appetite‐related proteins, including MC4R, POMC, and NPY, in the hypothalamus of the mice. Additionally, we measured the mRNA expression of three types of estrogen receptors: estrogen receptor (ER)α, ERβ, and the endoplasmic reticulum and plasma membrane‐localized estrogen receptor GPER. *Cyp19a1* exhibited significant positive correlations with *Esr1 and Mc4r* (Fig. 2A; *n* = 20). Furthermore, a significant correlation was observed between *Esr1* and *Mc4r* levels (Fig. 2B; *n* = 20).

### Ovarian estrogens did not directly affect the expression of Mc4r

**Fig. 3:**
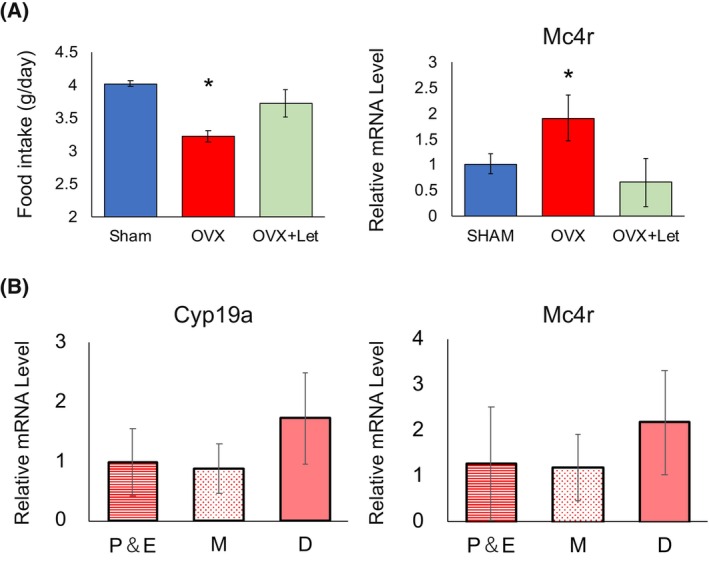
Food intake and *Mc4r* mRNA levels post‐OVX and letrozole treatment. (A) Comparative analysis of food intake and *Mc4r* mRNA levels in SHAM and OVX groups, and OVX mice treated with letrozole (20 mg·kg^−1^·mL^−1^ in corn oil) (OVX + Let), with control groups receiving corn oil only. (B) Expression levels of *Cyp19a1* and *Mc4r* mRNA in the hypothalamus across the phases P&E, M, and D. Statistical analysis was performed using one‐way ANOVA to evaluate differences among the groups. Tukey's *post hoc* test was conducted to identify pairwise differences between the groups. Results are presented as mean ± standard deviation (SD), and statistical significance was defined as *P < 0.05. All analyses were performed using spss.

Compared to SHAM mice, OVX mice exhibited reduced food intake; however, letrozole administration increased food consumption in OVX mice to a level comparable to that in SHAM mice and decreased *Mc4r* expression in the hypothalamus (Fig. 3A). In contrast, no changes were observed in the estrous cycle and *Mc4r* and *Cyp19a1* expression levels in the hypothalamus (Fig. 3B).

### ArKO mice showed decreased Mc4r expression and appetite upregulation

**Fig. 4:**
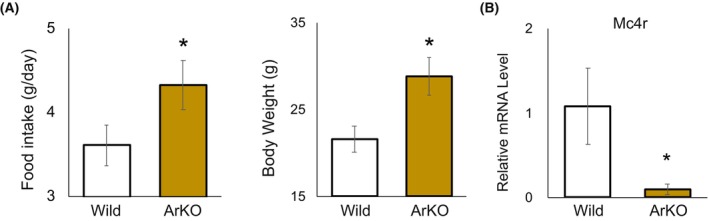
Impact of aromatase knockout on food intake and body weight. (A) Assessment of food intake and body weight in wild‐type (WT, *n* = 5) and aromatase knockout (ArKO, *n* = 5) female mice aged 12–15 weeks. (B) Expression level of Mc4r mRNA in wild‐type (WT, *n* = 5) and aromatase knockout (ArKO, *n* = 5) female mice aged 12–15 weeks. Student's *t*‐test was conducted to identify pairwise differences between the groups. Results are presented as mean ± standard deviation (SD), and statistical significance was defined as *P < 0.05. All analyses were performed using spss.

Female ArKO mice, lacking systemic estrogens, including neuroestrogens, demonstrated elevated body weight and increased food consumption, compared with the wild‐type mice (95‐day‐old wild‐type vs. ArKO mice: body weight, 21.6 ± 1.5 g vs. 28.9 ± 2.2 g; food intake, 3.7 ± 0.21 g vs. 4.5 ± 0.25 g; *P* < 0.05) (Fig. 4A). Moreover, *Mc4r* expression in the hypothalamus of ArKO mice was lower, compared to that in wild‐type mice (Fig. 4B).

### Estrogen in the culture medium and that synthesized in neurons increases Mc4r expression

**Fig. 5:**
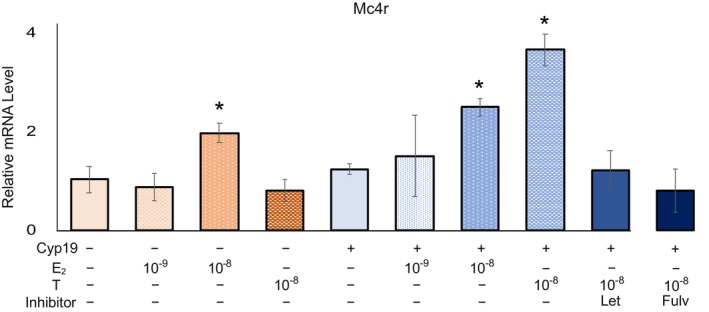
Effect of aromatase overexpression on *Mc4r* expression in hypothalamic neurons. Aromatase overexpression study in N38 hypothalamic neuron cell line (N38 O.E) with E_2_ or testosterone (T) at concentrations of 10^−9^ and 10^−8^ m, fulvestrant at concentrations of 10^−4^ m including an inhibition experiment with letrozole added to T 10^−8^ m in N38 O.E cells. DMSO served as the solvent control (*n* = 4). Statistical analysis was performed using one‐way ANOVA to evaluate differences among the groups. Tukey's *post hoc* test was conducted to identify pairwise differences between the groups. Results are presented as mean ± standard deviation (SD), and statistical significance was defined as *P < 0.05. All analyses were performed using spss.

To explore the significance of neuroestrogens synthesized by neurons themselves, we induced high levels of *Cyp19a1* expression in the mouse‐derived hypothalamic neuron cell line N38 (N38 overexpression; N38 O.E.), which initially did not exhibit any *Cyp19a1* expression. Under E_2_ 10^−9^ m (a concentration typical of serum from nonpregnant mice), *Mc4r* expression remained unaltered in both N38 and N38 O.E. However, at E_2_ 10^−8^ m, *Mc4r* expression significantly increased, induced by E_2_. While testosterone showed no effect on *Mc4r* expression in N38 cells, in N38 O.E cells, a notable increase in *Mc4r* expression was observed in response to testosterone (10^−8^ m). To determine whether this effect was due to E_2_ synthesized in N38 cells using testosterone as a substrate, the aromatase inhibitor letrozole was added. Our analysis showed that it did not affect *Mc4r* expression upon testosterone addition. In addition, the increase in *Mc4r* was abolished by fulvestrant, an ER inhibitor too (Fig. 5).

### Restoration of food intake in ArKO mice by brain‐specific aromatase gene guidance (BrTG‐ArKO)

**Fig. 6:**
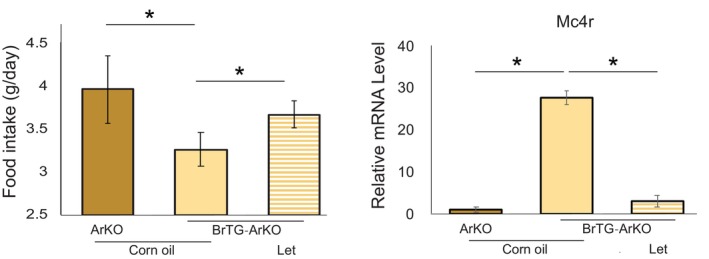
Food intake and *Mc4r* expression in ArKO and BrTG‐ArKO mice. Investigation of food intake and *Mc4r* mRNA levels in the hypothalamus of ArKO (*n* = 5) and brain‐specific aromatase‐restored BrTG‐ArKO (*n* = 5) female mice aged 12–14 weeks, with BrTG‐ArKO mice receiving Let (20 mg·kg^−1^) orally (*n* = 5). Statistical analysis was performed using one‐way ANOVA to evaluate differences among the groups. Tukey's *post hoc* test was conducted to identify pairwise differences between the groups. Results are presented as mean ± standard deviation (SD), and statistical significance was defined as *P < 0.05. All analyses were performed using spss.

The *Cyp19a1* gene comprises multiple exons, including the exon 1f, which is flanked with brain‐specific regulatory elements in the promoter region, and was specifically selected among the multiple exons of the gene. Previously, Harada *et al*. developed transgenic mice expressing human *CYP19a1* under the control of an exon 1f promoter sequence [19, 22]. These transgenic mice were subsequently crossed with ArKO mice to establish the BrTG‐ArKO line, characterized by the specific restoration of *Cyp19a1* expression in the central nervous system. Both ArKO and BrTG‐ArKO mice exhibited ovarian atrophy and serum estrogen levels below the limit of detection (Data not shown). Notably, compared to ArKO mice, BrTG‐ArKO mice demonstrated significantly reduced food intake and increased hypothalamic expression of *Mc4r* (Fig. 6).

### Neuroestrogens enhance leptin responsiveness

**Fig. 7:**
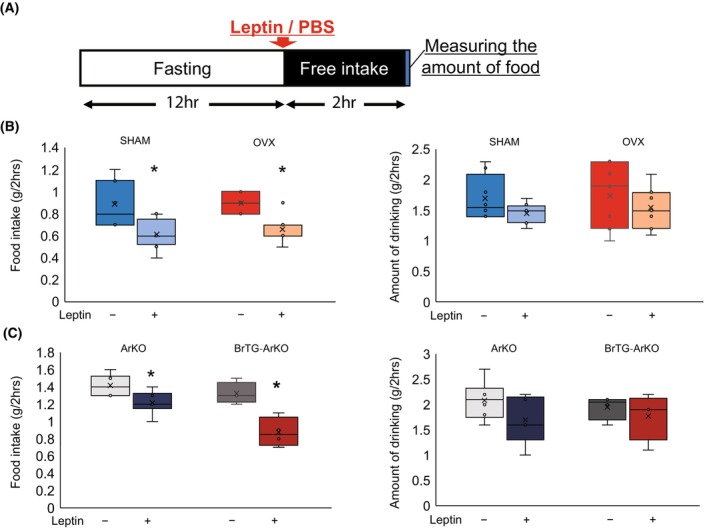
Leptin responsiveness in ArKO and BrTG‐ArKO mice. (A) Experimental setup for leptin administration in ArKO and BrTG‐ArKO mice. Mice were subjected to a 12‐h fasting period followed by a 2‐h feeding period to standardize their feeding schedule before intraperitoneal leptin injection. (B) Effect of leptin intraperitoneal administration on food and water consumption of SHAM and OVX group mice (*n* = 4). (C) Effect of leptin intraperitoneal administration on food and water consumption of ArKO and BrTG‐ArKO group mice (*n* = 4). All data are presented as mean ± SEM with significant differences was defined as *P < 0.05.

To compare the response of ArKO and BrTG‐ArKO mice toward leptin, the mice were fasted for 12 h, fed for 2 h to standardize their feeding schedule, and then administered leptin intraperitoneally (Fig. 7A). In the SHAM and OVX groups, food intake was significantly impacted. In the ArKO group, food intake was reduced after leptin administration (PBS vs. Leptin: 1.41 ± 0.44 vs. 1.27 ± 0.400). BrTG‐ArKO mice exhibited a greater leptin‐induced reduction in food intake (PBS vs. Leptin: 1.32 ± 0.109 vs. 0.88 ± 0.148), compared to ArKO mice (Fig. 7B,C).

## Discussion

This study explored the impact of ovariectomy on food intake and body weight, set against the backdrop of appetite‐suppressing properties of estrogen [27, 28]. OVX significantly decreased food intake compared to SHAM, a finding that initially seemed at odds with the role of estrogen as an appetite regulator (Fig. 1). Notably, despite a reduction in food intake observed in OVX mice aged 49–140 days, these mice exhibited a marked increase in body weight compared to their SHAM‐operated counterparts.

Our approach diverged from traditional ovariectomy methodologies, which are typically conducted in mice aged 8–10 weeks to coincide with the onset of ovulation [29]. By performing ovariectomy at 5 weeks, we aimed to minimize confounding variables associated with the maturation of the ovaries and subsequent estrogen fluctuations, thus focusing on the immediate effects postweaning. This modification revealed a significant decrease in food intake postearly ovarian ablation, suggesting an intricate interplay between estrogen levels and appetite regulation. The administration of letrozole, which increased appetite (Fig. 2D), hints at the critical role of local biosynthesis, particularly of neuroestrogen, in appetite suppression. This biosynthesis is likely facilitated by hypothalamic aromatase, underscoring the importance of neuroestrogens synthesized in the brain. This aligns with the finding that testosterone, via aromatase activity, promotes MC4R expression in neurons (Fig. 4). These results collectively point to a direct regulatory effect of neuroestrogens on appetite, distinguishing their impact from that of ovarian‐secreted estrogens, which are likely mediated indirectly by neuroestrogens.

Our research contributes to the evidence that neuroestrogens play a vital role in regulating appetite by enhancing the activity of MC4R. This was clearly the case even when there were problems with neuroestrogen production, as in the ArKO mice, or in cases where neuroestrogen production was specifically reinstated in certain neurons, as observed in BrTG‐Ar mice.

It is imperative to recognize the complex mechanism underlying appetite control, involving not only the hypothalamus, the central hub for appetite signals, but also other brain regions like the amygdala and the substantia nigra, which are associated with emotion and the reward system, respectively [30]. This complexity extends beyond a simplistic energy balance framework, suggesting that neuroestrogens may modulate appetite through multiple pathways, including those involved in emotional regulation [30].

The ovaries are the primary site of estrogen synthesis, and previous studies have demonstrated that estrogen regulates the expression of lipid‐metabolizing enzymes in adipose and hepatic tissues through endocrine actions. Furthermore, it has been shown that estrogen increases the expression of appetite‐regulating factors such as MC4R [31]. The regulatory region of the MC4R gene contains an ERα response element, and it has been demonstrated that estrogen increases MC4R expression through ERα‐mediated pathways [32]. In fact, studies using N38 O.E also showed that intracellularly synthesized neuroestrogens increased Mc4r via ER (Fig. 5), a result that confirms the studies using mice.

Additionally, leptin, secreted from adipose tissue, and lipocalin 2, secreted from bone tissue [33, 34], both have appetite‐suppressing effects. Although this study focuses on the role of neuroestrogen, it is believed that the mechanisms by which estrogen and neuroestrogen suppress appetite throughout the entire body are highly complex.

Estrogen mediates its effects primarily through ERα [2, 3], although the involvement of ERβ and G‐protein‐coupled estrogen receptors has also been noted [30, 35, 36]. Additionally, aromatase activity, and consequently neuroestrogen levels, are subject to dynamic changes and fluctuate within a 4–15 nm range [21], influenced by factors such as phosphorylation via calcium calmodulin‐dependent protein kinase type II [25, 37]. Our findings emphasize that neuroestrogens regulate key appetite‐related genes (*Pomc*, *Mc4r*, *Lepr*, and *Npy*) through various estrogen receptors, independent of systemic estrogen levels. Considering the potential modulation of intraneuronal aromatase activity by different neurotransmitters, we propose that the influence of neuroestrogens on appetite is characterized by significant complexity.

## Materials and methods

### Mice

Mice were bred under controlled conditions at 22 ± 2 °C and 55–65% humidity. Female 35‐day‐old C57Bl/6 mice (Chubu Kagaku Shizai Co., Ltd., Aichi, Japan) were subjected to either OVX or SHAM. ArKO mice were developed through the targeted disruption of the aromatase gene *Cyp19a1* in heterozygous mutant C57Bl/6 mice; their wild‐type counterparts served as controls. Each group, including OVX, SHAM, ArKO, and the wild‐type mice, comprised four animals. Body weight and food intake were monitored weekly until the mice reached 140 days of age.

To transiently inhibit estrogen synthesis, letrozole (Fujifilm Wako, Osaka, Japan) was suspended in corn oil (Fujifilm Wako) and orally administered at a dose of 20 mg·kg^−1^·day^−1^ per animal. Three groups, each consisting of four mice, were prepared for the experiment: SHAM, OVX, and OVX + Let containing OVX mice receiving only corn oil and letrozole. Each mouse was individually housed, and changes in body weight and daily food consumption were recorded every 7 days. Food consumption was calculated as one‐seventh of the total consumption (in grams) over the 7 days. All animal experiments were approved (APU22095) by the Degree Experimental Committee of Fujita Medical College; all procedures involving the mice were performed under anesthesia.

Recombinant mouse leptin (CYT‐351 Peptide Institute, Inc., Osaka, Japan) was dissolved in ice‐cold Tris/HCl buffer (20 mm, pH 8) to prepare a stock solution of 1 mg·mL^−1^. This stock solution was further diluted with ice‐cold phosphate‐buffered saline (PBS; pH 7.4) to obtain a working solution of concentration 0.3 mg·mL^−1^, as described previously [38]. The mice received vehicle intraperitoneal injections twice daily for 3 days to establish a feeding baseline, followed by intraperitoneal injections of leptin (3 mg·kg^−1^ body weight; stock leptin solution diluted in ice‐cold PBS, pH 7.4) or the vehicle (equivalent volume of 20 mm Tris/HCl diluted in ice‐cold PBS, pH 7.4).

### Vaginal smears and vaginal plugs

To confirm the sexual cycle, the vagina was washed with PBS, and regular vaginal smears were performed. After collecting the vaginal smear samples, they were dropped onto glass slides and stained with 4% Giemsa stain (Sigma‐Aldrich, St. Louis, MO, USA) for observation.

### Cell culture

The mouse‐derived hypothalamic neuron cell line N38 (RRID: CVCL_D438; Cellutions Biosystems Inc., Burlington, ON, Canada) was cultured in phenol red‐free RPMI 1640 medium containing 10% fetal bovine serum and treated with charcoal dextrin at 37 °C under 5% CO_2_ (Nichirei Biosciences Inc., Tokyo, Japan) and confirmed to be mycoplasma‐free throughout the culture process. E_2_, T, and Fulvestrant (Fujifilm Wako Pure Chemicals, Osaka, Japan) were added to the cells and dissolved in dimethyl sulfoxide (Fujifilm Wako Pure Chemicals) to a final concentration of 0.1%.

Mouse genomic DNA was used as a template, and the fragments were amplified using polymerase chain reaction. The brain‐specific promoter region of the mouse *Cyp19a1* gene was amplified using the following primer pairs: MB‐AR‐N1 (5′‐TCACTGTTCACAGAGTAC‐3′) and MB‐AR‐1R (5′‐GGACTCTTGAAGATGGTGAG‐3′). The mouse apolipoprotein AI promoter region was amplified using the mo‐apoA1‐2 (5′‐TGGACCCCTGGGAGTCTGC‐3′) and mo‐apoA1‐R1 (5′‐GGACGCTCTCCGACAGTCT‐3′) primers. The cDNA clones were subcloned into the p3XFLAG‐myc‐CMV‐26 expression vector (Sigma‐Aldrich) to obtain the pFLAG‐ARP‐1 expression plasmid. This plasmid was introduced into the N38 cells using Lipofectamine 3000 (Thermo Fisher Scientific, Waltham, MA, USA) to generate N38TG. Additionally, the experimental plans involving cell studies were approved by the Institutional Biosafety Committee (approval no. DP24012).

### Quantitative PCR

RNA was extracted from the brain samples of the mice, and the cellular mRNA expression level of the genes was determined using real‐time PCR. Total RNA was extracted using the PrimeScript RT reagent kit (TaKaRa Bio, Tsu City, Shiga, Japan). Quantitative PCR was performed in triplicate using the ABI Perkin‐Elmer Prism 7300HT Sequence detection system (Applied Biosystems, Foster City, CA, USA). TaqMan gene expression assays were used to detect the expression of aromatase (*Cyp19a1* Mm00484049_m1), *Lepr* (Mm00440181_m1), *Oxt* (Mm01329577_g1), *Oxtr* (Mm01182684_m1), *Mc4r* (Mm00457483_s1), *Npy* (Mm01410146_m1), and *Pomc* (Mm00435874_m1). *Gapdh* (Mm999999915_m1) was used as a housekeeping gene. Relative quantities were determined using the ΔΔ*C*_t_ method, according to the manufacturer's instructions.

### Statistical analysis

The statistical significance of differences in aromatase protein levels was analyzed with one‐way ANOVA. If differences were found to be significant, subsequent analyses with *post hoc t*‐tests with Bonferroni correction were performed. Other statistical comparisons were conducted with a two‐tailed unpaired *t*‐test. These tests were performed using SPSS (IBM, Armonk, NY, USA).

## Conflict of interest

The authors declare no conflict of interest.

## Author contributions

TH performed most of the experiments and wrote the paper. EN helped to develop the research plan and write the paper. Research funding was provided by TH and EN. KK, XH, and SN helped with breeding mice and ovariectomy. TK determined the mouse estrous cycle determination and drawing. NH and SH helped with the creation and breeding of genetically modified mice, as well as with the formulation of the research plan and the writing of the paper. YS discussed the results and supported the writing of the paper.

## Peer review

The peer review history for this article is available at https://www.webofscience.com/api/gateway/wos/peer‐review/10.1111/febs.17426.

## Data Availability

The data that support the findings of this study are available from the corresponding author upon reasonable request.
